# The Applications of Artificial Intelligence in Digestive System Neoplasms: A Review

**DOI:** 10.34133/hds.0005

**Published:** 2023-02-06

**Authors:** Shuaitong Zhang, Wei Mu, Di Dong, Jingwei Wei, Mengjie Fang, Lizhi Shao, Yu Zhou, Bingxi He, Song Zhang, Zhenyu Liu, Jianhua Liu, Jie Tian

**Affiliations:** ^1^ School of Engineering Medicine, Beihang University, Beijing, China.; ^2^ Key Laboratory of Big Data-Based Precision Medicine, Beihang University, Ministry of Industry and Information Technology, Beijing, China.; ^3^ CAS Key Laboratory of Molecular Imaging, Institute of Automation, Chinese Academy of Sciences, Beijing, China.; ^4^ Department of Oncology, Guangdong Provincial People's Hospital/Second Clinical Medical College of Southern Medical University/Guangdong Academy of Medical Sciences, Guangzhou, Guangdong, China.

## Abstract

**Importance:**

Digestive system neoplasms (DSNs) are the leading cause of cancer-related mortality with a 5-year survival rate of less than 20%. Subjective evaluation of medical images including endoscopic images, whole slide images, computed tomography images, and magnetic resonance images plays a vital role in the clinical practice of DSNs, but with limited performance and increased workload of radiologists or pathologists. The application of artificial intelligence (AI) in medical image analysis holds promise to augment the visual interpretation of medical images, which could not only automate the complicated evaluation process but also convert medical images into quantitative imaging features that associated with tumor heterogeneity.

**Highlights:**

We briefly introduce the methodology of AI for medical image analysis and then review its clinical applications including clinical auxiliary diagnosis, assessment of treatment response, and prognosis prediction on 4 typical DSNs including esophageal cancer, gastric cancer, colorectal cancer, and hepatocellular carcinoma.

**Conclusion:**

AI technology has great potential in supporting the clinical diagnosis and treatment decision-making of DSNs. Several technical issues should be overcome before its application into clinical practice of DSNs.

## Introduction

Digestive system neoplasms (DSNs) are the leading cause of cancer-related mortality worldwide [1– 3]. In 2020, 5 of the top 7 cancer types for estimated deaths belong to DSNs, including esophageal cancer, gastric cancer, colorectal cancer, hepatocellular carcinoma (HCC), and pancreas cancer [2]. Despite the fact that clinical treatment has improved, the prognosis of DSN patients is dismal, with a 5-year survival rate of less than 20% [4, 5]. Apart from the DSNs' aggressiveness, the unsatisfactory prognosis could be attributed to the dilemmas in reliable early diagnosis, accurate treatment response, and prognosis prediction [6– 8].

Tumor tissue-based genomic and proteomic technologies have shown the potential for precision medicine [9, 10]. However, these technologies suffer from the intrinsic limitation that molecular characterization from a small portion of tumor tissue could not represent the whole tumor due to the spatial and temporal heterogeneity of tumor [11, 12]. In contrast, medical imaging such as computed tomography (CT), magnetic resonance imaging (MRI), and positron emission tomography (PET) could provide a more comprehensive characterization of tumor and has been used in clinical routine for preoperative diagnosis and evaluation of treatment response.

Conventional radiological characteristics that originated from radiologists' experience, termed “semantic features”, are usually qualitative and subjective [13– 15]. Though useful in the preoperative diagnosis and treatment response evaluation, these features usually have large interobserver variability and limited predictive performance [13, 15– 17]. For example, Response Evaluation Criteria in Solid Tumors criteria and its revisions rely on the simple 1- or 2-dimensional size-based measurement of tumor, which have been questioned for its efficacy during the past years [18, 19]. In comparison, artificial intelligence (AI) algorithms could mine specific clinical task-related, high-dimensional, and quantitative features from medical images automatically [6, 20, 21], which could automate the complex process in the diagnosis or treatment response evaluation to assist clinicians and reduce their workload. It is noted that AI could mine features with powerful prediction value but could not be detected by humans visually, thereby improving the efficacy of clinical management [22– 25].

Here, we first introduce the AI algorithm for analyzing medical images and then review the representative applications of AI in the DSNs. Here, we focus on 4 common DSNs: esophageal cancer, gastric cancer, colorectal cancer, and HCC. Finally, we summarize the current challenge of AI and its potential directions for supporting clinical decision-making of DSNs.

## AI Methodology for Medical Image Analysis

**Fig. 1.**
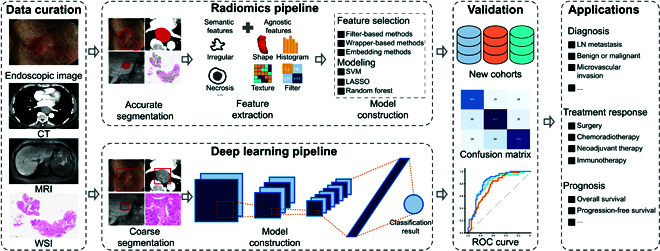
The AI analysis workflow in digestive system neoplasms. SVM, support vector machine; LASSO, the least absolute shrinkage and selection operator; LN, lymph node; ROC, receiver operating characteristic; AI, artificial intelligence.

AI is a technological science that studies and develops theories, methods, technologies, and application systems for simulating, extending, and expanding human intelligence, which was first introduced at a Dartmouth College conference in 1956 [26, 27]. In the last decade, AI has emerged as a promising approach for supporting clinical management [6, 20]. To achieve this, there are mainly 2 methods in AI: radiomics and deep learning. Both of them could convert medical images into quantitative features for characterizing tumor phenotype. The analysis workflow of radiomics and deep learning in DSNs includes data collection, region of interest (ROI) segmentation, feature extraction and selection, and model construction (Fig. 1).

### Data collection

Large-scale and multicenter datasets with appropriate annotations are usually needed for building a robust and generalizable AI model. However, the acquisition and reconstruction parameters of medical images, such as manufacture, voxel spacing, and reconstruction method, are usually varied among different hospitals. Those variations will induce changes in quantitative features and further influence the robustness and generalization of the AI model [28– 32]. There are several methods to mitigate the influence including image resampling [33, 34], image intensity normalization with or without references [28, 35], image augmentation including translation, rotation, flip, Gaussian blur [36, 37], and robust feature selection in terms of different parameters [38, 39]. In addition, details on imaging acquisition parameters and protocols should be reported in the AI-related studies for reproducibility and comparability. 

### ROI segmentation

After data collection, the subsequent analyses including ROI segmentation, feature extraction, and model construction are different between radiomics and deep learning. Radiomics analysis requires precise tumor segmentation by experienced radiologists. However, manual segmentation is labor-intensive and time-consuming and suffers from interobserver variability. Automatic or semiautomatic segmentation algorithms using convolutional neural network (CNN) are promising to solve these shortcomings. Common automatic segmentation algorithms include fully convolutional network, U-Net, Seg-Net, DeepLab, and their variations [40– 44]. Previous studies have shown that such algorithms could generate a satisfactory performance with a Dice coefficient larger than 0.9 for organ segmentation [45, 46], while their performance should be further improved for segmenting tumor regions, especially for DSNs [47, 48]. In contrast, deep learning analysis of medical images only needs a coarse segmentation of tumor region, such as a bounding box including the whole tumor region, which is easy to achieve and feasible to use in clinical practice. Apart from tumor region, several studies have also segmented and analyzed peritumoral regions and found additional predictive value of the peritumoral region [13, 17, 39, 49]. 

### Feature extraction

Both radiomics and deep learning could convert the segmented ROI into high-dimensional quantitative features, but in different manners. Radiomic features are designed manually and could be mainly divided into semantic and agnostic features [14]. Semantic features refer to the quantification of features that are visually obtained by radiologists, such as tumor shape, necrosis, and enhancement degree [13– 15]. Agnostic features are quantitative and could be extracted automatically from the segmented ROI according to the designed mathematical expressions and mainly include histogram-based features, shape- and size-based features, textural features, and filtered features [20]. To facilitate and standardize the extraction of agnostic features, van Griethuysen et al. [50] developed a flexible open-source platform termed Pyradiomics. Since then, plenty of radiomics studies extract radiomic features using Pyradiomics. Compared with radiomic features, deep learning features are learned automatically to characterize tumor phenotype using CNNs from the segmented ROI. Generally, the fully connected layer in the CNN can be regarded as deep learning features [51]. 

### Feature selection and model construction

High-dimensional quantitative features usually contain plenty of redundancy and irrelevance, which could cause overfitting during model construction [52, 53]. Therefore, feature selection should be performed for building a generalizable model. In radiomics, the most commonly used feature selection algorithms include (a) filter-based methods, such as univariate analysis, variance thresholding, and mutual information-based methods [23, 54, 55]; (b) wrapper-based methods, such as forward stepwise selection and recursive feature elimination [13, 56]; and (c) embedding methods, such as the least absolute shrinkage and selection operator (LASSO) and ridge regression [57, 58]. The combination of multiple feature selection methods in a sequential manner was also common in previous radiomics studies [16, 23, 39]. Considering the small sample size of data in some clinical situations, ensemble feature selection might be an effective approach for selecting more robust features [16, 23]. On the other hand, there are also several methods to avoid overfitting in deep learning, such as L1 and L2 norm regularizations and dropout [59]. Afterwards, a predictive or prognostic model was constructed based on the selected key features for predicting clinical outcomes. Radiomics usually utilizes machine learning algorithms to learn the linear or nonlinear mapping from key features to clinical outcomes. Commonly used machine learning algorithms in radiomics include support vector machine [60], LASSO [57], and random forest [61]. The optimal parameters in these algorithms are determined using cross-validation. CNN is the commonly used deep learning algorithm for analyzing medical images, such as ResNet [62], Xception [63], and DenseNet [64]. To train the model, a supervised learning modeling strategy that requires clinical labels for all training data was often utilized in radiomics or deep learning. 

## AI Applications in DSNs

**Fig. 2.**
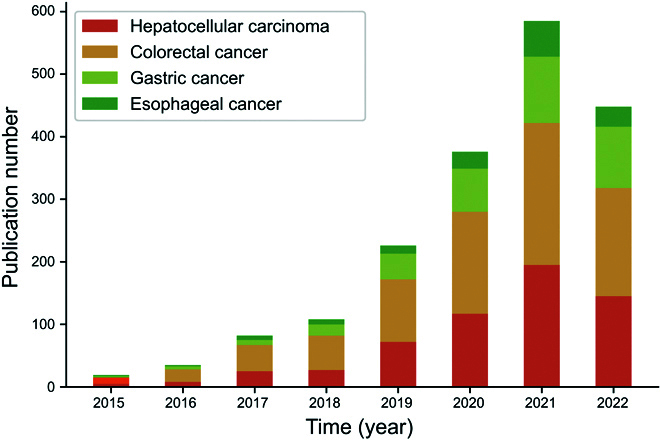
Statistics of AI-related studies including radiomics and deep learning in the 4 most common digestive system neoplasms. The total number of related publications is going straight up.

**Fig. 3.**
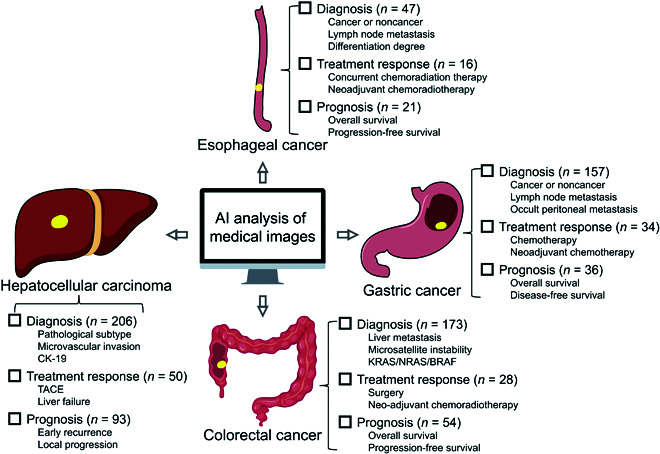
The representative applications of AI in digestive system neoplasms. AI, artificial intelligence ; TACE, transarterial chemoembolization; KRAS, Kirsten rat sarcoma viral oncogene homolog; NRAS, neuroblastoma rat sarcoma viral oncogene homolog; BRAF, v-Raf murine sarcoma viral oncogene homolog B.

In recent years, AI applications in DSNs have increased dramatically and have shown their potential for clinical application (Fig. 2). Here, we will describe the applications regarding diagnosis, evaluation of treatment response, and prognosis in the 4 most common DSNs: esophageal cancer, gastric cancer, colorectal cancer, and HCC (Fig. 3 and Table).

**Table.** Specifications of AI studies in the 4 most common digestive system neoplasms

**Studies**	**Study design**	**Patient number (training + validation)**	**AI methodology**	**Model architecture**	**Image modality**	**Clinical task**
**Esophageal cancer**						
Guo et al. [65]	Retrospective, multicenter study	549 + 2,123	Deep learning	SegNet	Endoscopy	Diagnosis
de Groof [67]	Retrospective and retrospective, multicenter study	414 + 255	Deep learning	U-Net	Endoscopy	Diagnosis
Takeuchi et al. [74]	Retrospective, multicohort study	411 + 46	Deep learning	VGG-16	CT	Diagnosis
Kawahara et al. [73]	Retrospective, single-center study	73 + 31	Radiomics	LASSO	CT	Diagnosis
Chen et al. [70]	Retrospective, single-center study	623 + 110	Radiomics	ANN	CT	Diagnosis
Qu et al. [72]	Retrospective, single-center study	90 + 91	Radiomics	Logistic regression	MRI	Diagnosis
Li et al. [75]	Prospective, multicenter study	203 + 103	Deep learning	ResNet	CT	Treatment response
Xu et al. [77]	Retrospective, single-center study	390 + 168	Deep learning	Multi-view multiscale CNN	CT	Treatment response
Beukinga et al. [81]	Retrospective, single-center study	139 + 60	Radiomics	Ensemble model	PET	Treatment response
Wang et al. [83]	Retrospective, single-center study	116 + 38	Radiomics + deep learning	Logistic regression + DenseNet-169	CT	Prognosis
Lin et al. [84]	Retrospective, single-center study	171 + 114	Deep learning	CNN with attention	CT	Prognosis
**Gastric cancer**						
Luo et al. [102]	Retrospective and prospective, multicenter study	15,040 + 69,390	Deep learning	DeepLab V3+	Endoscopy	Diagnosis
Hu et al. [106]	Retrospective, multicenter study	170 + 125	Deep learning	VGG-19	Endoscopy	Diagnosis
Dong et al. [17]	Retrospective, multicenter study	100 + 454	Radiomics	LASSO	CT	Diagnosis
Dong et al. [110]	Retrospective, multicenter study	225 + 505	Radiomics + deep learning	SVM + DesseNet-201	CT	Diagnosis
Cui et al. [124]	Retrospective, multicenter study	243 + 476	Radiomics + deep learning	LASSO + DesneNet-121	CT	Treatment response
Jiang et al. [121]	Retrospective, multicenter study	457 + 1,158	Deep learning	CNN	CT	Treatment response
Zhang et al. [125]	Retrospective, multicenter study	302 + 367	Radiomics + deep learning	Logistic regression, CNN	CT	Prognosis
Zhang et al. [126]	Retrospective, multicenter study	518 + 122	Deep learning	ResNet	CT	Prognosis
**Colorectal cancer**						
Huang et al. [130]	Retrospective, single-center study	326 + 200	Radiomics	LASSO	CT	Diagnosis
Zhou et al. [131]	Retrospective, single-center study	261 + 130	Radiomics	LASSO	MRI	Diagnosis
Liu et al. [132]	Retrospective, multicenter study	193 + 366	Radiomics	LASSO	CT + MRI	Diagnosis
Li et al. [133]	Retrospective, multicenter study	226 + 142	Radiomics	Logistic regression	CT	Diagnosis
Yamashita et al. [135]	Retrospective, multicenter study	115 + 479	Deep learning	MobileNetV2	WSI	Diagnosis
Liu et al. [55]	Retrospective, single-center study	152 + 70	Radiomics	SVM	MRI	Treatment response
Jin et al. [142]	Retrospective, multicenter study	481 + 141	Deep learning	Siamese network	MRI	Treatment response
Lu et al. [144]	Retrospective, multicenter study	502 + 526	Deep learning	Inception-v3 with RNN	CT	Treatment response
Shao et al. [146]	Retrospective, multicenter study	303 + 678	Radiomics	XGBoost	MRI + WSI	Treatment response
Feng et al. [147]	Retrospective and prospective, multicenter study	303 + 730	Radiomics + deep learning	SVM + VGG-19	MRI + WSI	Treatment response
Liu et al. [149]	Retrospective, multicenter study	176 + 453	Radiomics	LASSO-Cox	MRI	Prognosis
Liu et al. [150]	Retrospective, multicenter study	84 + 151	Deep learning	ResNet-18	MRI	Prognosis
Kather et al. [151]	Retrospective, multicenter study	86 + 934	Deep learning	VGG-19	WSI	Prognosis
**Hepatocellular carcinoma**						
Gao et al. [156]	Retrospective, multicenter study	612 + 111	Deep learning	CNN with RNN	CT	Diagnosis
Zhen et al. [157]	Retrospective, single-center study	1,210 + 201	Deep learning	Inception-ResNet V2	MRI	Diagnosis
Gu et al. [158]	Retrospective, multicenter study	364 + 91	Deep learning	DenseNet-121	CT	Diagnosis
Wei et al. [164]	Retrospective and prospective, multicenter study	635 + 115	Deep learning	ResNet-18	CT + MRI	Diagnosis
Xu et al. [161]	Retrospective, single-center study	350 + 145	Radiomics	SVM	CT	Diagnosis
Wang et al. [166]	Retrospective, single-center study	159 + 68	Radiomics	Decision tree	MRI	Diagnosis
Chen et al. [167]	Retrospective, multicenter study	111 + 33	Radiomics	Logistic regression	MRI	Treatment response
Liu et al. [169]	Retrospective, single-center study	491 + 246	Radiomics	SVM	CT	Treatment response
Peng et al. [171]	Retrospective, multicenter study	139 + 171	Deep learning	LeNet-5	MRI	Treatment response
Ji et al. [172]	Retrospective, multicenter study	210 + 260	Radiomics	Cox regression	MRI	Prognosis
Shan et al. [173]	Retrospective, single-center study	109 + 47	Radiomics	LASSO	CT	Prognosis

AI, artificial intelligence; LASSO, the least absolute shrinkage and selection operator; ANN, artificial neural network; CNN, convolutional neural network; SVM, support vector machine; RNN, recurrent neural network; CT, computed tomography; MRI, magnetic resonance imaging; PET, positron emission tomography; WSI, whole-slide image.

### Esophageal cancer

Esophageal cancer is a common malignancy, which includes 2 predominant subtypes: esophageal squamous cell carcinoma (ESCC) and esophageal adenocarcinoma [2]. Despite advances in treatment options, such as neoadjuvant chemoradiotherapy (NCRT) and immunotherapy, the prognosis of patients with esophageal cancer is dismal. Previous studies have shown that AI can help in the diagnosis and treatment response evaluation, thereby improving the prognosis of esophageal cancer patients [7, 65– 86]. 

#### Diagnosis

Endoscopic examination can help in diagnosing esophageal cancer at an early stage and is used in clinical routine. By quantitatively and automatically analyzing the endoscopic images, AI technologies could support the early diagnosis of esophageal cancer [7, 65– 69]. Liu et al. [7] developed a predictive model for distinguishing esophageal cancer from premalignant lesions using CNN. The developed model could achieve an accuracy of 0.858 on 1,272 white light endoscopic images from 748 esophageal cancer. Guo et al. [65] enrolled a multicenter retrospective cohort consisting of 6,473 narrow-band imaging images from 2,063 patients with precancerous and noncancerous lesions or ESCC. Based on these data, they developed a real-time computer-assisted diagnosis system for ESCC using a deep learning approach, which could achieve an area under the curve (AUC) of 0.989 and a sensitivity of 0.980. Furthermore, the computer-assisted diagnosis system could generate the probability heat map of cancerous lesion for each endoscopic image, which could assist in the early diagnosis of ESCC. In addition, deep learning has also shown its potential of early diagnosis of esophageal neoplasia in patients with Barrett's esophagus [66– 69].

Lymph node metastasis (LNM) status is closely associated with the prognosis of patients with esophageal cancer, and preoperative diagnosis of LNM can aid in treatment decision-making, such as extended lymphadenectomy and NCRT. Chen et al. [70] performed CT-based radiomic analysis of 733 patients with esophageal cancer for LNM prediction. The proposed radiomics model achieved an accuracy of 0.907 using artificial neural network. They also found that the predictive model using artificial neural network performed better than the one using logistic regression. The potential of radiomic approach for LNM prediction of esophageal cancer has also been demonstrated in other studies [71, 72]. In addition, radiomics-based approach could also be used for predicting differentiation degree [73] and distinguishing esophageal cancer from precancerous lesion [74]. AI models for LNM prediction in previous studies were only validated retrospectively; prospective validation should be performed in the future.

#### Treatment response and prognosis

Pretreatment evaluation of treatment response is also essential for esophageal cancer to aid individualized treatment decision-making. Plenty of studies have shown that quantitative imaging features from CT images could predict the treatment response including chemoradiation, immunotherapy plus chemotherapy, and concurrent chemoradiation therapy (CCRT) [75– 78]. However, the findings were only validated retrospectively in almost all studies expect the study by Li et al. [75]. In the study of Li et al., they developed an AI model for evaluating the treatment response to CCRT in locally advanced ESCC patients using a deep learning approach. The predictive model was developed and validated using a prospective and multicenter cohort from 9 Chinese hospitals and achieved a satisfactory predictive performance with an AUC of 0.833 in the validation cohort. NCRT was recommended for locally advanced resectable ESCC patients, and part of these patients could achieve pathological complete response (pCR) or local response. Recent studies confirmed that quantitative radiomic features from PET images have the potential for predicting NCRT treatment response including pCR, non-pCR, and local response [79– 81]. However, the predictive models developed in their studies should be validated further in a larger-scale and prospective cohort.

Besides treatment response, several AI-related studies focused on the prediction of prognosis. Larue et al. [82] found that radiomic features from CT images were associated with 3-year overall survival of patients with esophageal cancer. Wang et al. [83] found that combining radiomic and deep learning features from CT images could achieve better predictive performance for 3-year overall survival. Lin et al. [84] proposed a novel deep learning algorithm for overall survival prediction based on CT images, which achieved a better prognostic performance than using a radiomic approach or several common deep learning algorithms. The association between PET-derived radiomic features and prognosis was also confirmed in a few studies [85, 86].

### Gastric cancer

Gastric cancer is one of the most common cancers worldwide, with a high mortality rate [2]. The overall treatment effect of gastric cancer is poor. Timely and accurate diagnosis and individualized treatment are key to improving the survival rate and quality of life of patients with gastric cancer [87]. The National Comprehensive Cancer Network clinical practice guideline recommended noninvasive medical imaging technologies such as endoscopy, CT, MRI, and PET/CT as the main examination methods for the pretreatment diagnosis of gastric cancer patients [88]. As early as 2013, Ba-Ssalamah et al. [89] found that hand-crafted CT features could be used to distinguish gastric adenocarcinoma, gastric lymphoma, and gastrointestinal stromal tumor, suggesting that mining the CT texture patterns could quantify tumor heterogeneity. In the following years, many studies have been proposed to analyze the correlation between image features of gastric cancer and specific clinical problems, such as screening [90], staging [91– 94], Lauren classification [95, 96], Borrmann classification [97], treatment response [98, 99], and prognosis [100, 101]. These studies further laid the foundation for the development of AI analysis of gastric cancer images. At present, the applications of AI in image analysis of gastric cancer can be divided into diagnosis of categories and subtypes, and prediction of treatment response and prognosis. 

#### Diagnosis

Endoscopy is widely used to identify precancerous lesions and early gastric cancer lesions. Luo et al. [102] developed a real-time AI diagnosis system for upper gastrointestinal cancer based on more than 1 million white light endoscopy images from more than 80,000 patients. The system, modified from deeplab-V3 network architecture, could determine whether there were lesions in the input image and segment the suspected area in real time. Moreover, based on the white light endoscopy images, Wu et al. [103, 104] used CNN and deep reinforcement learning methods to develop an AI endoscopic diagnosis system called ENDOANGEL, and carried out a randomized controlled trial in 5 hospitals. They found that patients who received ENDOANGEL examination had fewer blind spots compared with the control group. The detection accuracy of the ENDOANGEL system was 84.7%, which was significantly better than the manual interpretation. However, correspondingly, the inspection time using the ENDOANGEL also increased slightly (5.40 vs. 4.38 min). In addition, they also developed an AI system for magnifying image-enhanced endoscopy [105]. Hu et al. [106] constructed and validated an AI model for the early diagnosis of gastric cancer based on the narrow-band images with magnifying endoscopy (ME-NBI) using deep learning. Based on the VGG-19 network pretrained on the public dataset ILSVRC-2012, they used transfer learning to fine-tune the model parameters. The gradient-weighted class activation mapping (Grad-CAM) [107] was used to visualize the ME-NBI area that the AI model focused on when making decisions. This technique could alleviate the poor interpretability of deep learning models, so as to enhance the trust of clinicians in the model. In addition, AI was also applied to evaluate the invasion depth of lesions, which was difficult for gastroscopy-based manual observation [108].

CT scan is the most commonly used noninvasive diagnostic technique for gastric cancer, especially for assessing local regional staging and distant metastasis. The development of various CT-based AI prediction systems for gastric cancer has become a research hotspot. Ma et al. [109] analyzed the venous phase CT images using radiomics and constructed a predictive model for identification of Borrmann type IV gastric cancer and primary gastric lymphoma, which achieved an AUC of 0.827. Dong et al. [17] proposed a predictive model for identifying occult peritoneal metastasis of gastric cancer based on CT image features of primary tumor and peritoneal microenvironment. On the multicenter validation cohorts, this model yielded an accuracy of more than 85% for patients with peritoneal metastasis who were previously missed by CT-based clinical diagnosis, indicating that it could reduce the risk of unnecessary surgical treatment for patients with occult peritoneal metastasis. This work has been referred by the guidelines for the diagnosis and treatment of gastric cancer published by the Chinese society of clinical oncology (CSCO) for 3 consecutive years (2019 to 2021). In addition, their team also used deep learning algorithms to predict other clinical indicators of gastric cancer, such as LNM [110], serosa invasion [111], and pathological type [112]. Aiming at predicting LNM of gastric cancer, Gao et al. [113], Liu et al. [114], and Meng et al. [115] used machine learning algorithm to build prediction models based on CT-derived radiomic features. Furthermore, Jin et al. [116] and Zhang et al. [117] constructed deep-learning-based prediction models for predicting LNM. The former model could analyze the metastasis of regional nodal stations one by one, and the latter model could output the segmentation results of lesions while predicting metastasis.

#### Treatment response and prognosis

Direct prediction of the treatment effect is expected to help clinicians in treatment decision-making for gastric cancer patients [118, 119]. Jiang et al. [120, 121] successively used radiomics and deep learning approaches to mine the imaging information of gastric cancer on PET/CT or CT images to predict the chemotherapy benefit of patients. The National Comprehensive Cancer Network guideline for gastric cancer recommended neoadjuvant chemotherapy (NAC) combined with surgery for the treatment of locally advanced gastric cancer [88]. However, clinical practice has found the obvious individual differences in NAC, and at least 20% of patients could not benefit. At present, researchers have constructed several NAC response prediction models using AI methods [122– 124] and achieved improved prediction performances. In addition, there is still a lack of high-precision clinical indicators to evaluate the prognosis of patients with gastric cancer. Although TNM staging is generally used as a reference to distinguish patients' risks, it is still necessary to further study biomarkers that are more directly related to prognosis. Many studies have shown that the AI prediction model could learn more deep-level survival imaging phenotypes by optimizing the design of deep learning networks [125– 127]. Zhang et al. [127] proposed a knowledge-guided multitask network. It enhanced the acquisition and use of key image features through the attention module and used the useful information contained in multitask learning to improve the prediction of survival risk. Jiang et al. [128] combined 2 clinical events, peritoneal recurrence and disease-free survival, and simultaneously predicted them through multitask learning to improve the network's ability of feature extraction and relationship mapping. Their model could effectively identify the high-risk patients who need intensive treatment.

### Colorectal cancer

Colorectal cancer is common and the second leading cause of cancer-related mortality worldwide [2]. AI has also been widely applied in colorectal cancer in recent years. 

#### Diagnosis

Preoperative prediction of LNM is able to aid in pretreatment decision-making, such as adjuvant therapy and lymph node dissection. Currently, MRI and CT are widely used in the diagnosis and staging of colorectal cancer patients in clinical practice [129], which objectively reflect tumor macroenvironment and depict tumor heterogeneity in a noninvasive approach. Huang et al. [130] analyzed the portal venous-phase CT images from 526 patients with colorectal cancer by radiomics and constructed a radiomics nomogram for clinical use, with an AUC of 0.778. The predictive value of MRI-based radiomic features for LNM was also confirmed [131]. Addressing the same clinical problem, Liu et al. [132] developed a predictive model based on both CT and MRI and found that the predictive model incorporating CT- and MRI-based radiomic features performed better than models based on radiomic features from either CT or MRI. In addition, CT-based radiomic features were also associated with microsatellite instability of colorectal cancer [133].

Pathological examination is the gold standard for colorectal cancer diagnosis. Different from CT and MRI, hematoxylin-and-eosin-stained whole-slide images (WSIs) can not only give a picture of the tumor microenvironment but also provide abundant microscopic information that could not be distinguished visually but could reflect molecular characteristics or heredity. Pathomics, like radiomics, has also achieved promising results in cancer diagnosis. Pathomics features were used to construct the model to improve the initial nodal staging in T3 rectal cancer [134]. Combined with radiomic features, the radiopathomics model has produced better prediction [134]. Besides, several studies have revealed that deep-learning-based models in histopathology have potential for predicting microsatellite instability [135, 136], the status of key molecular pathways and key mutations [137], and molecular subtypes [138], which could be an alternative and results in time and cost savings in clinical workflow.

#### Treatment response and prognosis

The development of various AI prediction models for evaluating the treatment response of NCRT in locally advanced colorectal cancer has attracted plenty of attention. MRI-based radiomic features were proved to be associated with post-NCRT treatment response including pathological complete/good response, local response, and no response [55, 139– 141]. For instance, Liu et al. [55] analyzed the T2 and DWI MR images from 222 colorectal cancer patients and proposed a radiomics-based AI model for predicting pCR, which achieved an AUC of 0.976 in the validation cohort. Deep-learning-based AI models for predicting treatment response of colorectal cancer patients were also developed [142– 145]. Lu et al. [144] analyzed serial CT images from 1,028 metastatic colorectal cancer patients and developed an AI model using deep learning for predicting early on-treatment response. They found that the AI model performed better than the tumor size change-based criteria, which is used in current clinical practice. Recently, researchers attempted to build a more accurate prediction model by incorporating multisource tumor images that could jointly improve the description of tumor heterogeneity. Some researchers have combined the characterization information of the macroscopic description of tumor heterogeneity with the characterization information of the microscopic description of tumor heterogeneity to construct an “image-pathology” description of tumor heterogeneity [146, 147]. They found that combining analysis of MRI and WSI could achieve better performance for predicting pCR in locally advanced rectal cancer. Predicting the treatment efficacy of colorectal cancer based on the AI analysis of medical images has also been recognized by experts and societies, and the study by Shao et al. [146] has been referred by the 2021 edition of the CSCO Colorectal Cancer Diagnosis and Treatment Guidelines.

In addition, AI has also made a successful attempt to predict local recurrence [148], disease-free survival [149– 152], and overall survival [148, 152, 153] based on retrospective cohorts with colorectal cancer patients. It helps to divide the risk of patients at an early stage, which, in turn, assists in precise treatment decisions and improves patient survival.

### Hepatocellular carcinoma

HCC accounts for almost 90% of primary liver cancers [2]. Along with the advances in AI technology in recent years, the potential of radiomics and deep learning methods has been proven for predicting diagnosis and prognosis of HCC. 

#### Diagnosis

Previous studies have shown that an AI-based diagnostic model for HCC has a superior accuracy and lower time cost compared to manual diagnosis and can also assist clinicians without much experience in improving the efficiency of diagnosis [154, 155]. Hamm et al. enrolled 494 hepatic lesions with multiphase MRI and developed a deep learning system for liver tumor diagnosis and found that their proposed system performed better than radiologists for classifying HCC (sensitivity: 90% versus 60% to 70%) [155]. Several studies showed that an AI model incorporating quantitative features from MRI and clinical data could provide a better diagnostic performance for HCC than models based on either MRI features or clinical data [156, 157]. Several studies also showed that AI could be used to predict the histological grade of HCC [158, 159].

Predicting microvascular invasion (MVI) of HCC using AI technology is another research hotspot. MVI is closely associated with posthepatectomy recurrence in HCC patients [160]. Preoperatively predicting MVI will help to develop a tailored surgical strategy. Radiomic features from contrast-enhanced CT images and MRI were shown to be closely related to MVI [161, 162]. Deep learning has also showed its potential in predicting MVI of HCC [163, 164]. Wei et al. [164] enrolled both contrast-enhanced CT images and gadoxetic-acid-enhanced MRI of HCC patients and developed an AI-based prediction model for MVI using a deep learning approach. They found that an AI model based on gadoxetic-acid-enhanced MRI performed better than that based on contrast-enhanced CT images.

In addition, MRI-based radiomics analysis can be used for predicting programmed cell death protein 1/programmed cell death protein ligand 1 expression [165] and cytokeratin 19 status [166], demonstrating the potential of MRI and AI techniques to extract noninvasive biomarkers.

#### Treatment response and prognosis

AI-based prediction of treatment response and prognosis can assist the selection of individualized treatments in HCC patients. Previous studies have demonstrated the potential of AI in predicting the liver failure of HCC patients after hepatectomy [167, 168] and treatment response of transarterial chemoembolization [169– 171]. Ji et al. [172] developed and validated an AI model for predicting recurrence risk of HCC patients after surgical resection based on 470 contrast-enhanced CT images from 3 independent institutions, which achieved better prediction performance than current staging systems. AI can also help to predict recurrence of HCC patients with ablation [173]. Furthermore, AI could also be used to predict the risk of HCC in chronic hepatitis B patients [174, 175] and detect the local tumor progression [176].

## Challenges and Future Opportunities

Current published studies have shown the great potential of AI in supporting clinical diagnosis and treatment decision-making of DSNs. However, there are several challenges to overcome before the application of AI into clinical practice of DSNs.

To train a robust and clinically applicable AI model, especially for the deep learning model, for a specific clinical problem, large-scale and well-annotated image data are usually needed. Though there are a large number of medical images of DSNs, well-annotated image data are limited. To alleviate such issue, a transfer learning approach is widely used currently: first, train an AI model on the publicly available ImageNet dataset consisting of over 14 million natural images with 1,000 classes [177] and then fine-tune its weight on the in-house medical imaging dataset. Nevertheless, due to the huge difference that existed between natural images and medical images, the weight of the AI model on natural images might not be suitable for medical images, which usually induced a suboptimal model for the given clinical task. Therefore, mining the valuable information from the easier collected unlabeled dataset is another choice. A semisupervised modeling strategy, such as mean teacher network [178], and a self-supervised modeling strategy, such as contrast learning [179], are usually used to make full use of both the large unlabeled dataset and the limited well-annotated dataset, which have shown improved predictive performance compared to models developed with only a well-annotated dataset using a fully supervised modeling strategy. At present, several published studies have developed and validated AI models for auxiliary diagnosis and treatment response prediction in DSNs based on large-scale and well-annotated image datasets [65, 102, 121]. These high-quality datasets, however, are usually not publicly available, which might hinder the validation and comparison of different AI models. Therefore, data sharing is vital for a robust and clinically applicable AI model.

At present, the majority of published studies on AI in DSNs rely on accurate, labor-intensive, and time-consuming ROI segmentation by radiologists, which might hinder its clinical application. Fully automatic segmentation algorithms based on deep learning hold promise to alleviate this issue and have achieved satisfactory performance for segmenting multiple organs [46], such as esophagus, liver, and stomach, among others. However, the automatic segmentation performance of DSN is limited due to the complexity of tumors [47]. On the other hand, modeling based on the whole organ images not only could avoid the accurate segmentation of DSNs but also might achieve better performance than modeling based on the tumor region due to the analysis of peritumoral microenvironment. Wang et al. [180] found that deep learning analysis of the whole lung could achieve better prediction performance for EGFR than analysis of the region of lung cancer.

Another limitation of such studies in DSNs is the interpretability of AI models, especially for deep learning models. Although the success of AI has been demonstrated in diagnosis and evaluating treatment response of DSNs, it is always questioned due to its black-box nature. Recently, several methods have been proposed to visualize the deep learning features and prediction models, such as CAM [181], Grad-CAM [107], and Ablation-CAM [182]. By visualizing where the trained AI model pays attention to, these methods could explain the model to some extent. In addition, almost all AI models in the published studies were validated using retrospective cohorts. Before their application into clinical practice, they should be validated in large-scale prospective cohorts from multiple centers.

Previous studies have shown that AI models incorporating both MRI and WSIs could perform better than models based on either MRI images or WSIs alone [146, 147]. This indicated that AI can aggregate multiple information, and incorporating more information into AI, such as radiographic images, pathologic images, genomics, proteomics, and diagnosis reports, might generate a more powerful predictive system.

In addition, AI mainly extracts quantitative features from reconstructed medical images, such as CT and MRI, and has achieved surprising results in a variety of clinical auxiliary diagnosis and treatment tasks in previous studies [149, 183, 184]. However, the image reconstruction procedure inevitably causes information loss, distortion, and variations among medical images, eventually leading to the irretrievable bias in the subsequent analysis. Therefore, the use of AI technology to directly construct the mapping from signals to knowledge has attracted the attention of some researchers [185– 188], which is expected to bring new breakthroughs for precision medicine. During this procedure, how to decouple the key features from large-size raw data is an essential technical problem that needs to be solved.

In conclusion, AI has shown its potential for aiding diagnosis and predicting treatment response and prognosis of DSNs through a large number of studies. However, several issues need to be overcome before its application into clinical practice of DSNs.

## Data Availability

No new data were created for this manuscript.
